# Transglutaminase-Catalyzed Glycosylation Improved Physicochemical and Functional Properties of *Lentinus edodes* Protein Fraction

**DOI:** 10.3390/foods12091849

**Published:** 2023-04-29

**Authors:** Shan-shan Wu, Wei Han, Yan-fen Cheng, Shao-jun Yun, Ming-chang Chang, Fei-er Cheng, Jin-ling Cao, Cui-ping Feng

**Affiliations:** 1 College of Food Science and Engineering, Shanxi Agricultural University, Jinzhong 030801, China; 2 Collaborative Innovation Center of Quality and Efficiency of Loess Plateau Edible Fungi, Jinzhong 030801, China; 3 Shanxi Key Laboratory of Edible Fungi for Loess Plateau, Jinzhong 030801, China

**Keywords:** *Lentinus edodes* protein fraction, TGase, glycosylation, physicochemical and functional properties

## Abstract

*Lentinula edodes* has high nutritional value and abundant protein. In order to develop and utilize edible mushroom protein, this study was designed to investigate the effects of TGase-catalyzed glycosylation and cross-linking on the physicochemical and functional properties of *Lentinus edodes* protein fraction. The results showed that within a certain time, glycosylation and TGase-catalyzed glycosylation decreased the total sulfydryl, free sulfydryl, disulfide bond, surface hydrophobicity, β-fold and α-helix, but increased the fluorescence intensity, random coil, β-turn, particle size and thermal stability. The apparent viscosity and the shear stress of the protein with an increase in shear rate were increased, indicating that TGase-catalyzed glycosylation promoted the generation of cross-linked polymers. In addition, the TGase-catalyzed glycosylated proteins showed a compact texture structure similar to the glycosylated proteins at the beginning, indicating that they formed a stable three-dimensional network structure. The flaky structure of proteins became more and more obvious with time. Moreover, the solubility, emulsification, stability and oil-holding capacity of enzymatic glycosylated *Lentinus edodes* protein fraction were significantly improved because of the proper TGase effects of glycosylation grafting and cross-linking. These results showed that glycosylation and TGase-catalyzed glycosylation could improve the processing characteristics of the *Lentinula edodes* protein fraction to varying degrees.

## 1. Introduction

*Lentinus edodes* protein fraction, extracted from *Lentinus edodes* via alkali dissolution and acid precipitation, is a mixture of proteins. *Lentinula edodes* protein fraction not only has good functional and physicochemical properties, such as foaming, emulsification, oil-holding capacity and thermal and pH stability, but also has anti-inflammatory, anti-tumor and immunomodulatory activities, which have potential application value in functional foods [1,2,3,4]. These properties of the protein can be improved via modification for elevating its nutritional potential and functional properties [5]. To promote the use of the protein, extensive studies have been carried out to improve its functional properties, such as the use of physical, chemical and biological enzymatic modifications [6,7,8].

Among these methods, glycosylation is the most common because it is effective for different proteins. By producing protein–polysaccharide conjugates, the Maillard reaction has been proven to enhance the functional properties of proteins, including solubility, foaming, emulsification, thermal stability and gelling [9]. For example, the properties of whey protein were improved to varying degrees after glycosylation treatment, and, in particular, the thermal stability of it increased by eight times compared with the untreated protein [10]. Moreover, after the glycosylation reaction of β-lactoglobulin and chitosan, its emulsifying properties were enhanced [11]. However, the application of the Maillard reaction in the food industry is limited, because of its uncontrolled high temperature conditions and its generation of toxic byproducts [12]. Enzymatic glycosylation, as a potential new method of large-scale glycosylation, has been widely studied owing to its site-specific modification and mild reaction conditions.

Transglutaminase (TGase) is a type of acyltransferase. It can catalyze acyl transfer reactions between gamma-carboxyamide groups of protein/peptide glutaminyl residues (acyl donors) and primary amines (acyl acceptors), leading to the formation of ε-(γ-glutamyl)-lysine cross-linking in proteins, as well as coupling amino-containing polysaccharides to proteins, to enhance certain properties of modified proteins [13]. Since it recognizes all kinds of primary amines, various conjugates can be produced by TGase-mediated acyl-transfer reactions and changes are induced in their structures and functional properties [14,15]. For example, treatment with TGase significantly increased the emulsification and the emulsifying stability of casein [16]. In addition, the functional properties of isolated leguminous proteins were improved after TGase cross-linking, especially the emulsification property [17]. Compared to non-enzymatic glycosylation, TGase-catalyzed glycosylation is considered an effective method for site-specific conjugation due to its amenability to mild reaction conditions and strict substrate specificity, and because of which the protein or peptide can be site-specifically modified; moreover, the toxic byproducts produced via the glycosylation pathways can disappear. 

Glucosamine (GlcN), a naturally occurring amino sugar, is a component of glycosaminoglycans in cartilage [18]. TGase can catalyze the amino group of GlcN to form an amide bond (-CO-NH-) with the gamma-amide group of Gln in the *Lentinus edodes* protein fraction and covalently bind to the protein [19]. Therefore, TGase can catalyze the glycosylation of protein to improve the biological activity and functional characteristics of food protein hydrolysates. However, there is no literature reported on the effects of TGase-catalyzed glycosylation on the physicochemical and functional properties of *Lentinus edodes* protein fraction.

Thus, we prepared *Lentinus edodes* protein fraction with different degrees of glycosylation and cross-linking via TGase-catalyzed glycosylation between *Lentinus edodes* protein fraction and GlcN. Furthermore, the effects of TGase-induced glycosylation and cross-linking on the physicochemical and functional properties of *Lentinus edodes* protein fraction were investigated, including sulfhydryl groups, surface hydrophobicity, fluorescence intensity, secondary structure, particle size distribution, apparent morphology, thermal stability, apparent viscosity and shear stress, as well as the solubility, emulsification, emulsion stability and oil-holding capacity. This study proposes a green and mild method for the glycosylation modification of *Lentinus edodes* protein fraction, which provides a reference for the development of the functional food of *Lentinula edodes* protein fraction and the comprehensive utilization of *Lentinula edodes*. 

## 2. Materials and Methods

### 2.1. Preparation of Lentinus Edodes Protein Fraction

*Lentinus edodes* fruit bodies were obtained from China Taigu Edible Fungi Engineering and Technology Center (Taigu, Jinzhong, China). The preparation of *Lentinus edodes* protein fraction referred to the method described by Fan et al. [20] with slight modifications. *Lentinus edodes* fruit bodies were dried and crushed to 150 mesh using an ultra-fine grinding vibration mill (WFM-10, Jiangyin Xiangda Machinery Manufacturing Co., Ltd., Jiangyin, China). The *Lentinus edodes* powder was mixed with distilled water in the ratio of 1:45. Then, the mixture was adjusted to pH 10.0 and put in a 50 °C incubator (HH-2, Saidelis Experimental Analyzer Instrument Factory, Tianjing, China) for 3 h. After centrifugation at 10,000 rmp for 15 min, the supernatant was collected and adjusted to the isoelectric point 4.2 of *Lentinus edodes* protein fraction with 1 mol/L HCl. Next, the solution was left to stand overnight at 4 °C to precipitate the protein. Afterwards, the solution after acid precipitation was centrifuged to obtain the sediment, which was washed with water until it was neutral and was freeze-dried in a vacuum freeze dryer (SHIA-10A-50A, CHRIST, Hagen, Germany) to obtain the *Lentinus edodes* protein fraction. The procedure was repeated three times to obtain about 13 g *Lentinus edodes* protein fraction from 100 g of *Lentinus edodes* powder.

### 2.2. TGase-Catalyzed Glycosylation of Lentinus Edodes Protein Fraction

The *Lentinus edodes* protein fraction was dissolved in distilled water (10.0% protein, *w/w*). The D-(þ)-Glucosamine hydrochloride (GlcN, SigmaeAldrich, St. Louis, MO, USA) and *Lentinus edodes* protein fraction (with a mass ratio of 1:1) were dissolved in 50 mL 10 mM phosphate-buffered solution (PBS, pH 7.0) with gentle stirring for 5 min to fully dissolve it. Next, the solution was adjusted to pH 7, sealed and put in a 50 °C constant-temperature-heating magnetic stirrer (RCT basic, IKA, Staufen, German) to heat for 30, 60, 90, 120 and 150 min, respectively. After the reaction, the sample was cooled in an ice water bath for 30 min, freeze-dried and stored at −18 °C for further experiments. 

The *Lentinus edodes* protein fraction was dissolved in distilled water (10.0% protein, *w/w*) and mixed with GlcN in a ratio of 1:1 in 50 mL 0.01 M phosphate-buffered solution (PBS, pH 7.0). The mixture was adjusted to pH 7 and then 8% guinea pig liver transglutaminase (EC 2.3.2.13) (TGase, SigmaeAldrich, St. Louis, MO, USA) was added to react in a 50 °C thermostatic water bath (DK-S10, Shanghai Boxun Industrial Co., Ltd., Shanghai, China) for 30, 60, 90, 120 and 150 min. After the TGase activity was inactivated at 90 °C for 10 min, the sample was freeze-dried in a vacuum freeze dryer (SHIA-10A-50A, CHRIST, Hagen, Germany) and stored at −18 °C for further use.

To remove the excess TGase and GlcN, collected aliquots were subjected to an ultrafiltration membrane system with molecular weight cut-offs of 100 kDa and 500 Da (Millipore Corporation, Billerica, MA, USA), respectively. Original *Lentinus edodes* protein fraction was used as the control. All prepared samples were stored at −40 °C until further analysis.

### 2.3. Physicochemical Properties

#### 2.3.1. Determination of Free Sulfydryl and Total Free Sulfydryl Group

The contents of free sulfydryl and total free sulfydryl were determined based on the principle that the sulfydryl group reacts with 5,5’-dithio bis nitrobenzoic acid (DTNB) to form a yellow compound with the maximum absorption peak at 412 nm using the non-protein sulfydryl content detection kit (BC1435, Beijing Solarbio Technology Co., Ltd., Beijing, China) and the total sulfydryl content detection kit (BC1375, Beijing Solarbio Technology Co., Ltd., Beijing, China). 

The contents of free sulfydryl and total free sulfydryl were calculated according to the following formulae:

Total sulfydryl content (μmol/g prot) = y × V ÷ (Cpr × V) = y ÷ Cpr(1)

Free sulfydryl content (μmol/g mass) = x × V ÷ W(2)

Note: y: concentration of standard tube (µmol/mL); V: volume of added extract (1 mL); Cpr: concentration of sample protein (mg/mL); W: sample mass (g).

#### 2.3.2. Determination of Surface Hydrophobicity

The determination of surface hydrophobicity was slightly modified based on a previous study [21]. The protein was dissolved in 10 mM PBS (pH 7.0) to a mass concentration of 5 mg/mL. Then, 200 μL of 1 mg/mL bromophenol blue solution was added to 1 mL of 5 mg/mL protein and centrifuged at 6000 rpm for 15 min. The supernatant was diluted 10 times and its absorbance was measured at 595 nm using a UV-Vis spectrophotometer (UV9100A, Beijing LabTech Co., Ltd., Beijing, China) with the phosphate buffer as the blank. The surface hydrophobicity was expressed by the binding amount of bromophenol blue.

The surface hydrophobicity was calculated according to the following formula:

Surface hydrophobicity (binding amount of bromophenol blue/μg) = 200 × (A_1_ − A_2_)/A_1_

where A_1_ is the absorbance of the phosphate-buffered solution and A_2_ is the absorbance of the supernatant.

#### 2.3.3. Intrinsic Fluorescence Emission Spectrum

The protein samples were dissolved in 0.01 M PBS (pH 7.0) to obtain the final concentration of 1 mg/mL. Then, the changes in the protein aromatic amino acid residues were determined using a Hitachi F4500 fluorometer (Tokyo, Japan) with an excitation wavelength of 298 nm and a scanning wavelength of 323~450 nm.

#### 2.3.4. Fourier Transform Infrared (FTIR) Spectroscopy

The 1.0 mg dry sample was mixed with 200 mg potassium bromide in the mixing mortar and the secondary structure was measured using an FTIR spectrometer (Madison Nicolet Is 10, Thermo Nicolet Co., Waltham, MA, USA) at a full wavelength (4000–400 cm^−1^) with the ambient temperature at 25 °C, the wave number at 4000~400 cm^−1^ and a resolution of 4 cm^−1^. Based on a previous study, the PeakFit v4.04 software was used for the baseline correction, Gaussian deconvolution and second derivative fitting [22]. Afterward, the assignment of each peak was measured, and the secondary structural elements (α-helix, β-sheet and β-turn) were quantified in each group.

#### 2.3.5. Determination of Particle Size

The 2 mg/mL protein solution was obtained by mixing the sample and 0.01 mM PBS (pH 7.0). The particle size distribution and the average particle size were determined using a particle size analyzer (HL2020-B, Beijing Haixinrui Technology Co., Ltd., Beijing, China) [23]. Each sample was repeated three times.

#### 2.3.6. Scanning Electron Microscopy (SEM) Analysis

The freeze-dried protein fraction was struck on a copper pile with a double-sided carbon label and coated with platinum. Then, the changes in surface microstructure were observed using a JSM-6490LV SEM (JEOL Electronics Co., Ltd., Tokyo, Japan) and photographed under an acceleration voltage of 15 kV.

#### 2.3.7. Analysis of Apparent Viscosity

The MCR 102 rheometer was used to determine the rheological property of 8% protein fraction samples [24]. The test conditions were as follows: 25 ± 0.1 °C, 50 mm, 1° lamina, 0.6 mL of protein solution and 0.103 mm of intervertebral space. Under the linear scanning mode, the steady shear rate varied from 0 to 100 s^−1^. The variation in shear stress with shear rate was measured. 

The steady rheological fitting curve of the protein solution was fitted linearly using the Herschel–Bulkley model τ = τ HB+ c γ P, where τ HB represents yield stress, c is the viscosity coefficient, p is the flow characteristic index and γ is the shear rate [7]. 

#### 2.3.8. Thermal Stability

The thermal properties of *Lentinus edodes* protein fraction were measured using a differential scanning calorimeter (DSC, 821 e, Mettler Toledo, Zürich, Switzerland). About 0.4 mg of *Lentinus edodes* protein fraction powder was put into a 40 μL aluminum pan and the pans were hermetically sealed using an aluminum cap. After being equilibrated at 28 °C for 2 h, the thermal properties were recorded during the heating scan from 25 °C to 470 °C with a constant heating rate of 10 °C/min and a nitrogen flow rate of 40 mL/min, using an empty pan as a reference. The temperature at maximum heat flow, the peak denaturation temperature (T d) and enthalpy change (ΔH) were detected from the thermograms using the Stare Software (Version 8.10, Mettler Toledo, Switzerland). 

### 2.4. Processing Properties 

#### 2.4.1. Determination of Grafting Density

The grafting density was determined using the o-phthalaldehyde (OPA) method (Fayle et al., 2001). A total of 40 mg OPA was dissolved in 1 mL methanol, and then 2.5 mL of 20% (*w/v*) sodium dodecyl sulfate (SDS, Solarbio Technology Co., Ltd, Beijing, China), 25 mL of 0.1 mol/L barax and 100 µL of β-mercaptoethanol were added. Then, the mixture was diluted to 50 mL using distilled water, which was called OPA reagent. Next, 4 mL OPA reagent and 200 µL sample solution were mixed and reacted at 35 °C for 2 h to measure the absorbance value (A) at 340 nm using a UV-Vis spectrophotometer (UV9100A, Beijing LabTech Co., Ltd., Beijing, China). The mixture of 4 mL OPA reagent and 200 µL distilled water was set as the control sample. The calculation formula is as follows:

DS(%) = (A_0_ − A_t_)/A_0_ × 100

DS: grafting degree; A_0_: absorbance of sample before grafting reaction; A_t_: absorbance of sample after grafting reaction.

#### 2.4.2. Determination of Solubility

The sample was diluted with distilled water to the final concentration of 5 mg/100 mL and shaken in a whirlpool at room temperature. After centrifugation at 4000 rmp for 10 min, the supernatant was used to determine the protein content using the Coomassie brilliant blue method [25].

The protein solubility was calculated via the following formula:

Solubility (%) = (Supernatant [protein content/Total protein content) × 100

#### 2.4.3. Determination of Emulsibility and Emulsion Stability

The emulsibility and emulsion stability were determined according to a previous method [26]. The sample was diluted to 1 g/100 mL using distilled water. Then, 8 mL of 1 g/100 mL sample and 2 mL soybean oil were mixed and homogenized using a high shear dispersion emulsifier (QWL750CY, Shanghai Quanjian Mechanical and Electrical Co., Ltd., Shanghai, China) for 5 min. Next, the 1 mL emulsion was mixed well with 2 mL of 0.1% SDS solution to determine its absorbance value at 500 nm via a UV-Vis spectrophotometer (UV9100A, Beijing LabTech Co., Ltd., Beijing, China). The 0.1% SDS solution was set as the control.

The emulsibility and emulsion stability were calculated according to the following formula:

EC = (2 × two point three zero three × A_0_ × DF)/(C × ρ × θ × 10,000) (3)

EC: emulsification (m^2^/g); A_0_: absorbance of sample; DF: dilution ratio (100); ρ: optical path (1 cm); θ: proportion of oil phase.

Emulsification stability was determined according to the above method by putting the above-determined emulsion in room temperature for 10 min.

The emulsification stability was calculated according to the following formula:

ES (%) = (EC10/EC) × 100(4)

ES: emulsification stability (%); EC: emulsification capacity (m^2^/g); EC10: emulsification capacity after 10–10 min (m^2^/g).

#### 2.4.4. Determination of Oil-Holding Capacity (OHC)

First, a 0.2 g sample was put into a dry centrifuge tube and weighed (m_1_). Then, the sample was mixed with 5 mL soybean oil and centrifuged at 4000 rpm for 30 min to discard the supernatant. Next, the centrifuge tube and the sediment were weighed (m_2_). 

The oil-holding capacity was calculated based on the following formula:

OHC (%) = [(m_2_ − m_1_)/m] × 100

OHC: oil-holding capacity; m: mass of sample (g); m_1_: mass of sample and centrifuge tube (g); m_2_: mass of sample precipitation and centrifuge tube (g).

### 2.5. Statistical Analysis 

The data were expressed as mean ± standard deviation (n = 3). SPSS 23.0 was used to analyze the differences between treatments using one-way analysis of variance (ANOVA) followed by Dunnett’s multiple comparisons. Before analysis, the normal distribution and homogeneity of variance were determined using the Kolmogorov–Smirnov one-sample test and Levene’s test, respectively. No violation of the assumptions for ANOVA was detected. Significant differences between groups were expressed at *p* < 0.05. 

## 3. Results

### 3.1. Effects of Different Treatments on Physicochemical Properties of Lentinus edodes Protein Fraction

#### 3.1.1. Sulfydryl and Disulfide Bond

The sulfydryl and disulfide bonds are important for protein structural stability, and the conversion of them can cause the protein to agglutinate [27]. As shown in Table 1, compared with the control group, with the extension in glycosylation time, the total sulfydryl, free sulfydryl and disulfide bond showed a first decreasing and then increasing trend. The same trend was obtained for protein treated with enzymatic glycosylation. Under the same conditions of 8% TGase and 120 min, the total sulfydryl and free sulfydryl in the protein fraction treated with enzymatic glycosylation decreased, respectively, by 21.49% and 20.61%, compared with glycosylation (*p* < 0.05). This may be due to the enzymatic glycosylation reaction, which makes the protein form polymers and changes the structure of the protein, so that the sulfydryl group on the surface is converted or embedded [28]. Under the same conditions (8% TGase and 120 min), compared with glycosylation, the disulfide bond of enzymatic glycosylation was relatively unchangeable, which is because the steric hindrance between proteins caused by long sugar chains or excessive sugar hinders the formation of disulfide bonds [28].

#### 3.1.2. Surface Hydrophobicity

In polar solutions, surface hydrophobicity has a crucial impact on the stability, conformation and functional properties of proteins. Typically, the surface hydrophobicity of a protein after glycosylation modification is remarkably reduced [24]. Similarly, in this study, the addition of GlcN alone or in combination with TGase decreased the surface hydrophobicity of the protein fraction (Table 1). GlcN has good hydrophilicity and is rich in hydroxyl (-OH) [29]. Therefore, the reduction in the surface hydrophobicity of glycosylated *Lentinus edodes* protein fraction after glycosylation may be owing to the steric hindrance effects of covalently bound GlcN molecules or the reaction between sugar and protein, which can introduce some hydrophilic groups, reducing the surface hydrophobicity and increasing the solubility. Similar results were obtained in the binding of β-lactoglobulin and glucose, and the binding of soybean protein and arabic [30,31].

#### 3.1.3. Intrinsic Fluorescence Spectrum Analysis

**Figure 1:**
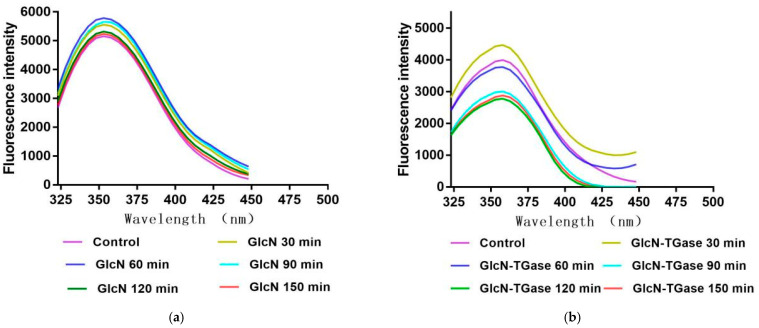
Effects of different treatments on fluorescence intensity of *Lentinus edodes* protein fraction. Note: (**a**) fluorescence intensity of *Lentinus edodes* protein fraction treated with GlcN; (**b**) fluorescence intensity of *Lentinus edodes* protein fraction treated with GlcN-TGase.

The protein tertiary structure is distinguished using intrinsic fluorescence spectroscopy via the exposure of fluorescing amino acids. In protein molecules, the hydrophobic amino acids (tryptophan, tyrosine and phenylalanine) that transmit fluorescence possess the maximum emission wavelength at an excitation wavelength of 290 nm [32]. Glycosylation treatment could significantly increase the fluorescent intensity of *Lentinus edodes* protein fraction (Figure 1a), which indicates that tryptophan is exposed to a more polar micro-environment. These phenomena confirm that GlcN successfully induces the conformational change in the *Lentinus edodes* protein fraction. Moreover, the redshift in the maximum fluorescence emission wavelength may be due to the fact that the tryptopphan group inside the protein is in a more hydrophilic environment, thus elevating the functional properties of *Lentinus edodes* protein fraction [33].

The fluorescence intensity of the protein treated with enzymatic glycosylation for 30 min was higher than that of the control (Figure 1b), which was caused by the low reaction degree at the beginning and the relative small molecular weight. However, with the extension in the reaction time, the spatial steric hindrance of oversized molecules has a shielding effect on the luminous group [34]; thus, the fluorescence intensity of the protein fraction treated with enzymatic glycosylation for 60, 90, 120 and 150 min decreased, which indicates that the glycosylation and cross-linking reactions between *Lentinus edodes* protein fraction and GlcN changed the polarity of tryptophan residues with the increase in TGase action time. This further proves that the addition of TGase not only promotes the glycosylation reaction, but also promotes the amino groups in amino sugars to enter the protein [35]. In addition, the red shift in the maximum fluorescence emission wavelength indicates that the structure of lysine residue has changed, the hydrophilicity of which has become larger and the solubility has also increased [33].

#### 3.1.4. Secondary Structure

FTIR spectroscopy is one of the tools used to analyze the chemical structure of a sample, on the basis of the absorption of the wavelength and the intensity of IR radiation. It can analyze the structural conformation of proteins via the examination of different absorption peaks of proteins in the infrared region [36]. The amide one band at 1700–1600 cm^−1^ in the infrared spectrogram is the area with the greatest change in the secondary structure of the protein, which is caused by the stretching vibration of C=O in the protein [37]. To clearly understand the stretching and aggregation properties of the protein, the secondary structures of the protein before and after treatment were investigated via the analysis of the planar bending of NH, the change in the -CN length in the amide one band (1700–1600 cm^−1^) region of the IR spectrum and the change in the C=O length in the structure [38].

As shown in Table 2, the β-turn was dominant in the control group. After treatment with glycosylation, the β-turn content was still the highest. With an extension in glycosylation time, the proportion of the β-folding and α-helix decreased first and then increased, which reached the lowest value after treatment for 120 min. However, the proportion of random coil and β-turn showed a first increasing and then decreasing trend, and reached the highest values of 29.53% and 49.93% when treated for 120 min, indicating that the protein structure grafted with sugar changed from order to disorder, and became more loose and flexible, which can effectively improve the functional properties [39].

The β-turn was still dominant in the protein treated with enzymatic glycosylation. The content of the random coil significantly increased compared to that of glycosylation. With the extension in the enzymatic glycosylation time, the β-fold and α-helix decreased first and then increased, and, respectively, reached the lowest value at 90 min or 120 min. The proportion of random coil and β-turn showed a first decreasing and then increasing trend, and reached the highest value of 31.38% and 38.14% when treated for 90 min. Moreover, the main structures of the protein became β-fold and random coil when treated for 120 min, which indicates that the enzymatic glycosylation reaction changes the structure of the protein from order to disorder, proved by the hydrophobic structure of the protein.

#### 3.1.5. Particle Size

**Figure 2:**
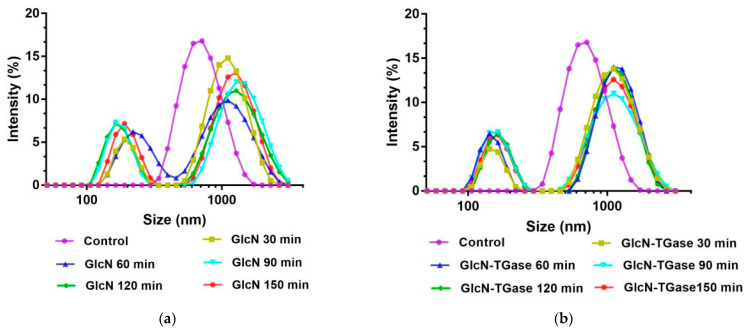

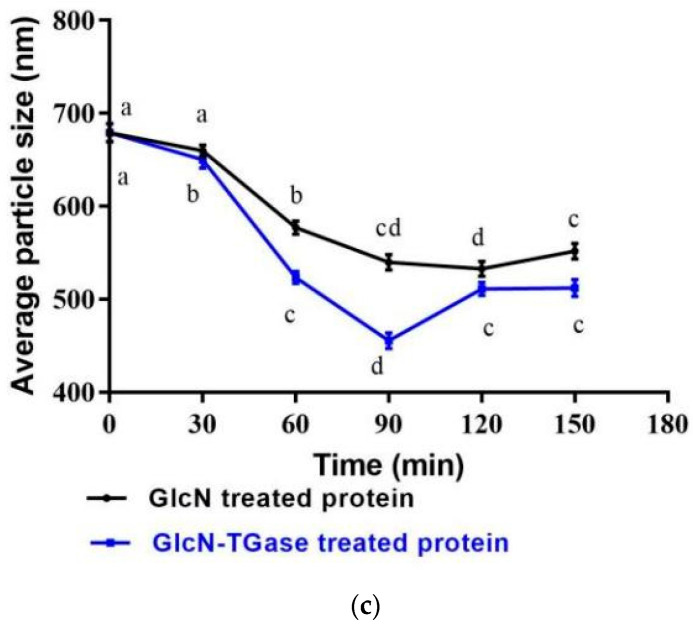
Effects of different treatments on particle size distribution and average particle size of *Lentinus edodes* protein fraction. Note: (**a**) particle size distribution of *Lentinus edodes* protein fraction treated with GlcN; (**b**) particle size distribution of *Lentinus edodes* protein fraction treated with GlcN-TGase; (**c**) average particle size of *Lentinus edodes* protein fraction treated with GlcN or GlcN-TGase. For the same index, different lowercase letters indicate significant differences between data (*p* < 0.05).

The particle size of the protein may affect the formation and stability of aggregates in protein solutions, thereby affecting the functional properties of proteins [40]. The control protein had only one single peak and the distribution was relatively uniform (Figure 2a). However, the glycosylated protein had two peaks, which was due to the glycosylation reaction between sugar and the protein to produce glycoprotein compounds, causing the structure of the protein molecules to curl and changing their dispersion [41]. With an extension in the time, the large particles gradually increased, indicating that the degree of glycosylation becomes higher and higher and the protein conformation changes with the introduction of monosaccharide molecules, and also indicating that the particle size will increase [42]. The particle size of the protein treated with enzymatic glycosylation showed a similar trend to that of glycosylation (Figure 2b), which was distributed around 1000 nm and 200 nm. This indicates that the reaction between TGase and GlcN makes the product more evenly distributed. These phenomena indicate that TGase catalyzes protein glycosylation and the cross-linking reaction to increase the size of protein aggregates, which agrees with a previously published report [43]. 

The average particle size of the protein treated with enzymatic glycosylation was higher than that of the untreated *Lentinus edodes* protein fraction (Figure 2c). However, under the same condition of 8% TGase and 120 min, the average particle size of the protein treated with enzymatic glycosylation was significantly reduced by 4% compared with glycosylation, indicating that enzymatic glycosylation would produce more small molecules while producing more stable compounds. 

#### 3.1.6. Microstructure

**Figure 3:**
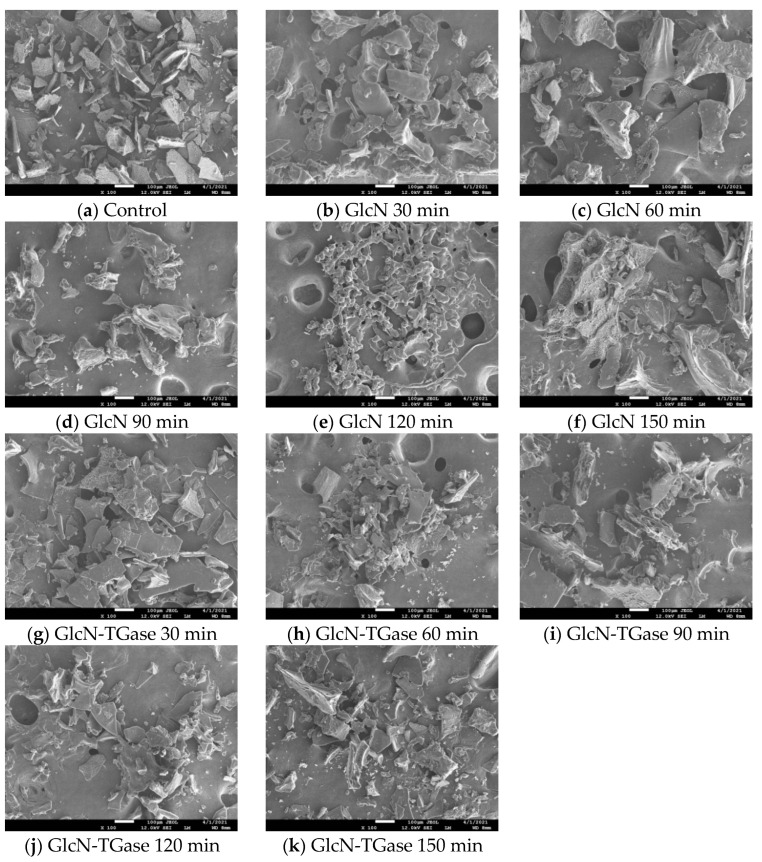
Effects of different treatments on the microstructure of *Lentinus edodes* protein fraction (magnification of SEM: 100×). Note: Control: *Lentinus edodes* protein fraction; GlcN 30 min: *Lentinus edodes* protein fraction treated with GlcN for 30 min; GlcN 60 min: *Lentinus edodes* protein fraction treated with GlcN for 60 min; GlcN 90 min: *Lentinus edodes* protein fraction treated with GlcN for 90 min; GlcN 120 min: *Lentinus edodes* protein fraction treated with GlcN for 120 min; GlcN 150 min: *Lentinus edodes* protein fraction treated with GlcN for 150 min; GlcN-TGase 30 min: *Lentinus edodes* protein fraction treated with TGase-catalyzed glycosylation for 30 min; GlcN-TGase 60 min: *Lentinus edodes* protein fraction treated with TGase-catalyzed glycosylation for 60 min; GlcN-TGase 90 min: *Lentinus edodes* protein fraction treated with TGase-catalyzed glycosylation for 90 min; GlcN-TGase 120 min: *Lentinus edodes* protein fraction treated with TGase-catalyzed glycosylation for 120 min; GlcN-TGase 150 min: *Lentinus edodes* protein fraction treated with TGase-catalyzed glycosylation for 150 min.

The surface of the control protein fraction was relatively smooth with small and disordered sheet structures (Figure 3a). After glycosylation treatment (Figure 3b–f), the surface of the protein fraction became more smooth, resulting in a relatively tight texture. It was likely that the covalent binding between the protein and sugar group caused the disappearance of small-molecular-weight polymers and an increase in the formation and content of large-molecular-weight protein polymers within and between protein molecules, leading to a tighter network structure [44]. With an extension in reaction time, the continuous reaction between GlcN and protein makes GlcN become wrapped on the surface of the protein, which weakens the hydrophobic interaction between molecules, and makes the graft unfold [45], proved by the solubility and graft rate of the protein. When treated for 120 min, the reaction between GlcN and the protein reached the highest value, and the binding sites of GlcN and the protein reached saturation. If the reaction time is too long, there will be certain steric hindrance and overreaction, which will reduce the binding rate of the protein and GlcN.

At the beginning, the TGase-catalyzed glycosylated proteins showed a compact texture structure like with the glycosylated proteins, indicating that they formed a stable three-dimensional network structure (Figure 3g,k). With an extension in the time, the flaky structure of proteins became more and more obvious, which may be due to the mutual promotion of the TGase and glycosylation reaction, weakening the structure of protein and making the protein flaky. Thus, TGase promotes GlcN to enter the *Lentinus edodes* protein fraction and reduces the average particle size. However, the TGase-catalyzed glycosylated protein was smaller and less rigid compared with the untreated *Lentinus edodes* protein fraction and GlcN-treated *Lentinus edodes* protein fraction. Therefore, TGase can promote the combination of GlcN and *Lentinus edodes* protein fraction to decrease the particle size and significantly enhance the protein’s flexibility [45].

#### 3.1.7. Apparent Viscosity

Shear strain sweeps are generally used to reflect the ability of the interface to resist the shear of the tested sample [46]. As shown in Table 3, the R^2^ of all samples was above 0.99, which indicates that the *Lentinus edodes* protein fraction solution treated with glycosylation and enzymatic glycosylation conforms to the Ostwald flow model. This showed a shear thinning pseudoplastic fluid after treatment for less than 60 min. When treated for 60 min, the *Lentinus edodes* protein fraction presented a shear thickening swelling fluid. Additionally, the *Lentinus edodes* protein fraction treated with enzymatic glycosylation showed a shear thickening swelling fluid. With the increase in shear rate, the apparent viscosity of the glycosylated *Lentinus edodes* protein fraction and TGase-catalyzed glycosylated *Lentinus edodes* protein fraction decreased, and then it remained stable after 80 1/S. It is proposed that when the shear rate is raised (0–80 s^−1^), the intermolecular force is destroyed, and the motion resistance of the molecular chain is reduced, thus decreasing the apparent viscosity produced by the orientation resistance. When the shear rate exceeds a certain threshold, the molecular chain of the system forms a relatively stable and orderly structure, and the change in shear rate will not affect the apparent viscosity.

Moreover, the proteins treated with different methods showed obvious differences. When the shear rate reached 30 1/S, the apparent viscosity of the glycosylated *Lentinus edodes* protein fraction decreased slowly. In addition, at a certain shear rate, the apparent viscosity of the glycosylated protein fraction was higher than that of the control, which indicates that the reaction between the protein and sugar increases the molecular size of *Lentinus edodes* protein fraction, thereby increasing the molecular volume [47]. The increase in particle size will increase the apparent viscosity.

The apparent viscosity of the TGase-catalyzed glycosylated *Lentinus edodes* protein fraction decreased with an increase in shear rate. At a certain shear rate, when the treatment time was within 90 min, the apparent viscosity of the protein fraction treated with enzymatic glycosylation was significantly elevated compared with the control and the glycosylated protein. This is because the glycosylation reaction catalyzed by the TGase covalently combines protein with glucose, increasing the interaction between proteins in the reaction system and thus increasing the apparent viscosity [7]. However, when the treatment time reached 120 min, the apparent viscosity was lower than that of the control protein, which may be due to the degradation of the polymers or the formation of steric hindrance, owing to the excessive reaction time. It can also be seen from the microstructure that the treatment for 120 min makes the protein flake, which reduces the apparent viscosity.

**Figure 4:**
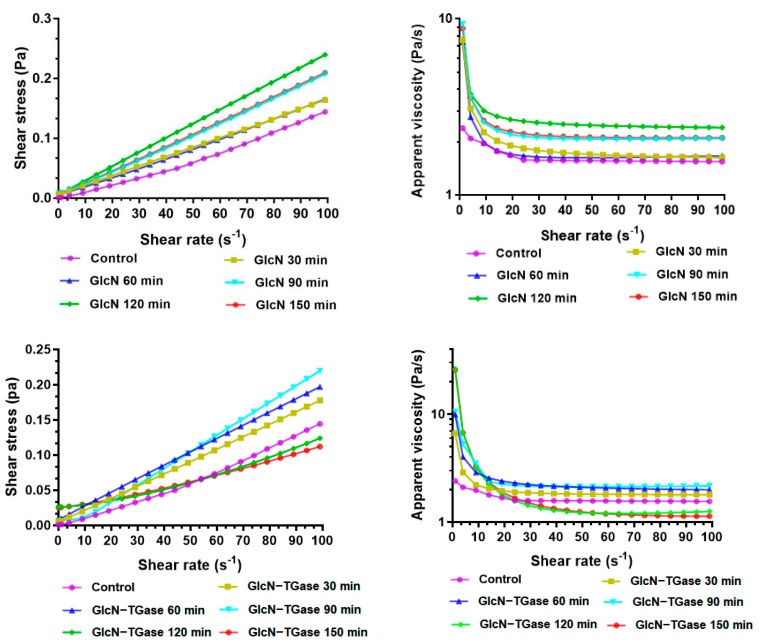
Effects of different treatments on rheological properties of *Lentinus edodes* protein fraction.

As shown in Figure 4, the shear stress of the protein fraction after treatment increased with an increase in shear rate. The glycosylation reaction between sugar and the protein makes the hydroxyl group of sugar be added to the protein to strengthen the hydrogen bond force. Moreover, the TGase promotes the covalent reaction between the protein and glycosyl, and the force of the covalent bond during which is greater than that of the hydrogen bond; thus, the shear stress of the enzymatic glycosylation treatment is higher than that of glycosylation in a certain time range.

#### 3.1.8. Thermal Stability

Thermal denaturation temperatures (Td) and ΔH are two prominent parameters in describing the thermal properties of a sample and can, respectively, reflect thermal stability and the extent of an ordered structure. As shown in Table 4, the denaturation temperature and enthalpy value of the protein after treatment show an increase. The Td value and enthalpy value of the control protein were 196.09 ± 1.01 °C and 2.34 ± 0.21 J/g, respectively. After treatment with glycosylation, the Td value and enthalpy value increased, respectively, to 306.85 ± 1.98 °C and 35.20 ± 0.37 J/g, which indicates that the covalent binding of glycosyl and the protein inhibits the interaction between proteins, or that the introduction of hydrophilic sugar groups changes the structure of the protein by ordering the molecules, enhancing the internal force and maintaining the integrity and connectivity of the protein network structure, thus increasing the thermal stability through covalent bonds [48].

After enzymatic glycosylation, the Td value and enthalpy value increased to 426.06 ± 2.52 °C and 116.37 ± 0.92 J/g, which indicates that the enzymatic glycosylation, combined with the cross-linking of TGase, constantly maintains the good thermal stability of the protein by enhancing the force between or within the protein molecules, changing the spatial conformation and maintaining the three-dimensional network structure of the protein.

### 3.2. Effects of Different Treatments on Functional Properties of Lentinus edodes Protein Fraction

#### 3.2.1. Grafting Degree

**Figure 5:**
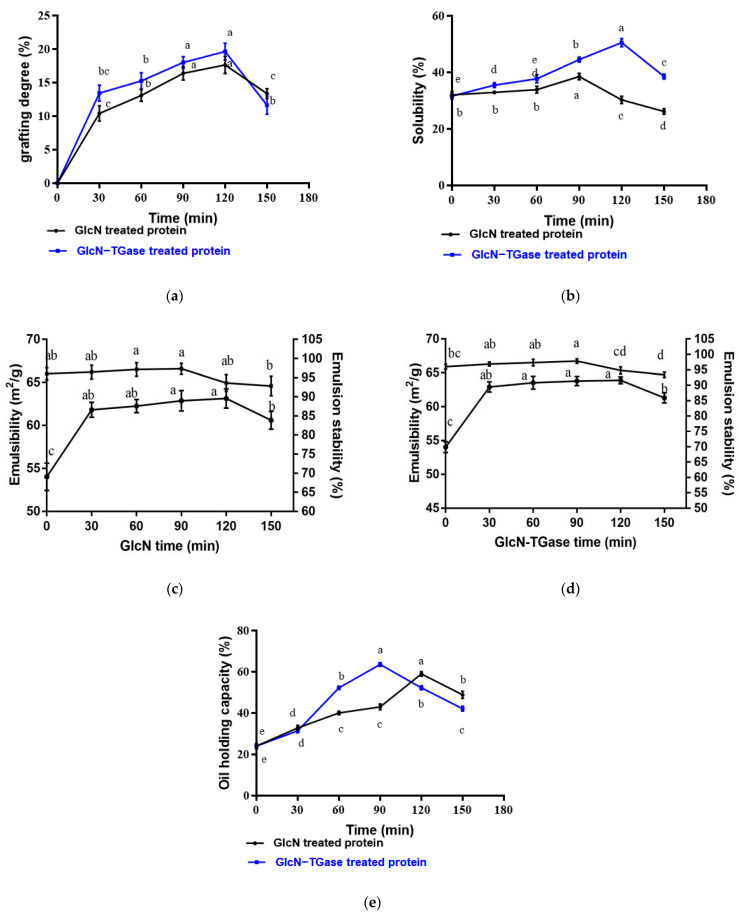
Effect of different treatments on the functional properties of *Lentinus edodes* protein fraction. Note: (**a**) grafting degree; (**b**) solubility; (**c**) emulsibility and emulsion stability treated with GlcN; (**d**) emulsibility and emulsion stability treated with GlcN-TGase and (**e**) oil-holding capacity. For the same index, different lowercase letters indicate significant differences between data (*p* < 0.05).

With an extension in the reaction time, the grafting rate of glycosylated protein fraction increased significantly (*p* < 0.05) and reached the maximum value at 120 min (Figure 5a). The essence of the Maillard reaction is the covalent combination of carbonyl ammonia. Thus, with an extension in the reaction time, free amino acids of the protein are exposed continuously, which makes the grafting rate continuously increase [49]. However, after 120 min, the grafting rate of the glycosylated protein began to decrease, which was consistent with a previous study [8]. The decrease in grafting rate may be caused by the re-polymerization of the glycosylated protein, owing to the participation of more amino groups in the reaction or owing to the partial degradation or structural changes in the graft product [50].

The grafting rate of the protein fraction treated with TGase-assisted glycosylation also showed a first increasing and then decreasing trend (Figure 5a). Moreover, the grafting rate of the protein after TGase-assisted glycosylation was higher than that of glycosylation. This may be because the TGase has a certain role in promoting glycosylation and can further promote the consumption of free amino acids [51].

#### 3.2.2. Solubility

High water solubility is of vital importance in proteins aimed to be used in the food industry, because solubility affects functional properties, such as thickening, emulsification and gelation [52]. With an extension in glycosylation reaction time, the solubility of the glycosylated protein showed the same trend as the grafting rate and reached the maximum value of 40.4% at 90 min (Figure 5b), which was consistent with the result of Li et al. [53]. Generally speaking, protein solubility can reflect the changes in the balance of surface hydrophobicity/hydrophilicity induced by glycosylation [54]. The protein solubility can be affected by the surface hydrophobicity of proteins, as confirmed by the reduced surface hydrophobicity of the glycosylated *Lentinus edodes* protein fraction. Therefore, the improved solubility of the glycosylated *Lentinus edodes* protein fraction might be owing to the attachment of hydrophilic GlcN moieties to the hydrophobic surface of protein molecules, resulting in enhanced solubility [55]. At the same time, the GlcN moieties can further impose an additional steric hindrance by preventing protein molecule aggregation, thus improving protein solubility [56,57]. After 90 min, the thermal polymerization between proteins decreases the protein solubility [55].

The same trend was observed in the solubility of TGase enzymatic glycosylation. The highest solubility was 50.5% at 120 min, which was consistent with a previous study [58]. However, under the condition of 8% TGase and 120 min, the solubility of the protein treated via enzymatic glycosylation was significantly increased by 18.26% compared with that of glycosylation (*p* < 0.05). TGase promotes the introduction of glucose glycosyl into the proteins, and the hydrophilicity of its hydroxyl group can further effectively improve the solubility of the protein [7]. Moreover, the covalently introduced glycosyl may further exert an additional steric hindrance effect to prevent the protein molecules from gathering near the isoelectric point, thereby improving the solubility of the protein [8].

#### 3.2.3. Emulsibility and Emulsion Stability

Emulsibility and emulsion stability, the important indexes for the emulsifying properties of proteins, are essential for their roles as food ingredients in food processing. With an extension in the glycosylation reaction time, the emulsibility and emulsion stability in GlcN-treated proteins showed first increasing and then decreasing trends, reaching the maximum value of 63.14 m^2^/g at 120 min and 97.34% at 90 min, respectively (Figure 5c,d). When the sample is glycosylated by GlcN and linked to the protein molecule via a covalent bond, the hydrophilic group of GlcN will attach to the protein fraction. When the hydrophobic group of the protein enters the oil phase, the hydrophilic group of GlcN will also enter the water phase. The amphiphilic covalent polymer has a strong adsorption force at the oil–water interface in order to form a stable hydrophilic lipophilic group, thereby enhancing the emulsion stability [17]. Moreover, the cationic groups added to the GlcN could inhibit the condensation of oil droplets in the lotion, and thus avoid the stratification of the system [59]. After 120 min, the emulsibility decreased, which was related to the decrease in the grafting products. After 90 min, the glycosylated products could not provide enough space stability, which led to a decrease in the emulsion stability. In addition, after the covalent binding of *Lentinus edodes* protein fraction and GlcN, the hydrophilic ability of the glycosylated *Lentinus edodes* protein fraction was elevated, making it an amphiphilic structure. This was in accordance with the SEM result that the formation of three-dimensional network structures contributes to an increase in the contact area with water and oil, thus improving the foaming and emulsifying properties of proteins, as well as other functional properties.

The trends of the emulsibility and emulsion stability in proteins treated with TGase-assisted glycosylation were consistent with those in proteins treated with glycosylation, reaching the maximum of 63.90 m^2^g^−1^ at 120 min and 97.82% at 90 min, respectively. The significantly improved emulsification properties are attributed to two effects. First, as shown in the results of particle size distribution, the addition of TGase and GlcN resulted in the formation of large aggregates, which increased the number of protein polymers on the water–oil interface per unit area and reduced the surface tension of the water–oil interface [60]. Second, the cross-linking of GlcN forms a network structure after the action of TGase, thereby effectively preventing aggregation between droplets [61]. When the processing time exceeds 120 min and 90 min, the cross-linking degree of GlcN and the protein will be reduced, and the emulsibility and emulsion stability will also be reduced [29].

#### 3.2.4. Oil-holding capacity

The oil-holding capacity plays a vital role in formulated food, which can measure the oil-binding capacity of non-polar side chains of proteins [62]. With the extension in glycosylation time, the oil-holding capacity increased first and then decreased, reaching a maximum value of 58.9% at 120 min (Figure 5e). A similar result was obtained in a previous study [63]. The exposure of hydrophobic groups in the protein via glycosylation can improve the oil-holding capacity of the products [64]. The decrease in the oil-holding capacity after 120 min may be due to the destruction of the three-dimensional network structure of the protein and sugar.

With the extension in the enzymatic glycosylation time, the oil-holding capacity also increased first and then decreased, reaching a maximum value of 63.6% at 90 min. A previous study showed that the oligosaccharide glycoslated and TGase cross-linked proteins exhibited higher oil-holding capacity [35]. In this study, under the condition of 8% TGase and 120 min, the oil-holding capacity of enzymatic glycosylation increased by 7.83% compared with glycosylation. This may be because the enzymatic glycosylation will make protein molecules form loose three-dimensional network structures to expose more oil-binding sites and intercept more oil molecules, thus improving the oil-holding capacity [65]. 

## 4. Conclusions

The present study systematically investigated the effects of TGase-catalyzed glycosylation on the physicochemical and functional properties of *Lentinus edodes* protein fraction. TGase-catalyzed glycosylated protein was mainly in a polymer format with reduced total sulfydryl, free sulfydryl, disulfide bond, surface hydrophobicity, β-fold and α-helix, and elevated fluorescence intensity, random coil, β-turn, particle size and thermal stability. With an increase in time, the enzymatic glycosylation effectively improved the apparent viscosity and shear stress with an increase in shear rate. Furthermore, TGase-catalyzed glycosylation cross-linking endows the *Lentinus edodes* protein fraction with higher solubility, as well as emulsifying and oil-holding capacity. These results can provide a theoretical basis for the processing and utilization of *Lentinus edodes* protein fraction. 

## Figures and Tables

**Table 1:** Effects of different treatments on the sulfhydryl group, disulfide bond and surface hydrophobicity of *Lentinus edodes* protein fraction.

Time (min)	Sulfydryl		Free Sulfydryl		Disulfide Bond		Surface Hydrophobicity	
	GlcN	GlcN + TGase	GlcN	GlcN + TGase	GlcN	GlcN + TGase	GlcN	GlcN + TGase
0	5.06 ± 0.09 ^a^	15.06 ± 0.09 ^a^	10.56 ± 0.11 ^a^	10.56 ± 0.11 ^a^	2.25 ± 0.07 ^a^	2.25 ± 0.07 ^a^	45.07 ± 0.93 ^a^	45.07 ± 0.93 ^a^
30	10.83 ± 0.06 ^b^	9.58 ± 0.16 ^b^	6.66 ± 0.10 ^b^	6.76 ± 0.04 ^b^	2.11 ± 0.05 ^a^	1.41 ± 0.10 ^b^	43.78 ± 0.20 ^ab^	45.06 ± 0.98 ^a^
60	4.88 ± 0.09 ^e^	5.33 ± 0.03 ^d^	4.01 ± 0.11 ^c^	4.53 ± 0.16 ^c^	0.44 ± 0.09 ^c^	0.39 ± 0.08 ^d^	36.77 ± 1.00 ^c^	41.64 ± 0.60 ^b^
90	4.46 ± 0.35 ^e^	4.72 ± 0.12 ^e^	3.94 ± 0.07 ^d^	3.97 ± 0.12 ^d^	0.26 ± 0.04 ^d^	0.37 ± 0.11 ^d^	37.65 ± 1.40 ^c^	38.48 ± 0.88 ^c^
120	5.77 ± 0.06 ^d^	4.53 ± 0.11 ^e^	4.28 ± 0.26 ^d^	3.39 ± 0.11 ^e^	0.75 ± 0.11 ^bc^	0.57 ± 0.12 ^d^	35.60 ± 1.41 ^c^	38.01 ± 1.38 ^d^
150	7.36 ± 0.06 ^c^	6.81 ± 0.06 ^c^	5.59 ± 0.39 ^d^	4.57 ± 0.06 ^c^	0.88 ± 0.16 ^b^	1.12 ± 0.01 ^c^	41.37 ± 1.22 ^b^	32.32 ± 0.84 ^d^

Note: for the same index, different lowercase letters indicate significant differences between data (*p* < 0.05).

**Table 2:** Secondary structure content of *Lentinus edodes* protein fraction after different treatments.

Protein	Secondary Structure Content/%			
	α-Helix	β-Fold	β-Turn	Random Coil
Control	13.97 ± 0.35 ^a^	29.37 ± 1.31 ^a^	32.97 ± 2.25 ^c^	23.69 ± 2.03 ^c^
GlcN 30 min	11.17 ± 0.26 ^a^	26.95 ± 1.64 ^b^	34.69 ± 1.87 ^c^	27.19 ± 1.98 ^b^
GlcN 60 min	10.00 ± 0.33 ^b^	24.39 ± 1.23 ^b^	38.01 ± 2.73 ^ab^	27.61 ± 1.64 ^b^
GlcN 90 min	8.39 ± 0.35 ^c^	23.91 ± 2.01 ^c^	39.00 ± 2.19 ^a^	28.70 ± 1.83 ^ab^
GlcN 120 min	6.97 ± 0.12 ^c^	20.54 ± 1.11 ^c^	42.93 ± 2.17 ^a^	29.56 ± 1.44 ^a^
GlcN 150 min	7.00 ± 0.15 ^c^	26.91 ± 1.53 ^b^	38.31 ± 2.18 ^b^	27.78 ± 1.21 ^b^
GlcN-TGase 30 min	10.40 ± 0.28 ^a^	22.39 ± 1.23 ^c^	36.12 ± 2.14 ^b^	31.09 ± 2.53 ^bc^
GlcN-TGase 60 min	10.01 ± 0.19 ^a^	21.75 ± 1.42 ^d^	36.52 ± 2.55 ^a^	31.72 ± 2.02 ^a^
GlcN-TGase 90 min	9.00 ± 0.19 ^a^	21.48 ± 1.54 ^d^	38.14 ± 1.96 ^a^	31.38 ± 1.89 ^ab^
GlcN-TGase 120 min	4.21 ± 0.06 ^b^	20.35 ± 1.19 ^d^	40.13 ± 2.11 ^b^	35.31 ± 1.23 ^b^
GlcN-TGase 150 min	5.99 ± 0.09 ^b^	22.71 ± 1.52 ^d^	36.01 ± 2.13 ^a^	35.29 ± 1.56 ^a^

Note: for the same index, different lowercase letters indicate significant differences between data (*p* < 0.05).

**Table 3:** Effects of different treatments on rheological properties of *Lentinus edodes* protein fraction.

Samples	K	N	R^2^
Control	0.004213	0.9285	0.99743
GlcN 30 min	0.005921	0.9886	0.99400
GlcN 60 min	0.006355	1.0939	0.99097
GlcN 90 min	0.007915	1.0691	0.99651
GlcN 120 min	0.005075	1.0137	0.99593
GlcN 150 min	0.005082	1.0235	0.99216
GlcN-TGase 30 min	0.007132	1.0408	0.99532
GlcN-TGase 60 min	0.007790	1.0263	0.99584
GlcN-TGase 90 min	0.008607	1.1098	0.99421
GlcN-TGase 120 min	0.026241	1.5105	0.99776
GlcN-TGase 150 min	0.025493	1.2657	0.99512

Note: K: viscosity coefficient; N: flow behavior index.

**Table 4:** Effects of different treatments on thermal properties of *Lentinus edodes* protein fraction.

Sample	T_0_ (°C)	T_d_ (°C)	ΔH (J/g)
Control	191.64 ± 1.21	196.09 ± 1.01	2.34 ± 0.21
GlcN-treated protein	287.72 ± 1.91	306.85 ± 1.98	35.20 ± 0.37
GlcN-TGase-treated protein	346.45 ± 2.01	426.06 ± 2.52	116.37 ± 0.92

Note: for the same index, different lowercase letters indicate significant differences between data (*p* < 0.05).

## Data Availability

All original data included in this paper are available from the authors upon reasonable request.

## References

[1] Wang Z.H., Fan X.Z., Yao F., Yin C.M., Shi D.F., Gao H., Shen W.Y. (2023). Extraction, characteristic analysis and amino acids evaluation of *Lentinula edodes* protein. Modern Food Sci. Technol..

[2] Erjavec J., Kos J., Ravnikar M., Dreo T., Sabotiˇc J. (2012). Proteins of higher fungi-from forest to application. Trends Biotechnol..

[3] Hibino Y., Konishi Y., Koike J., Tabata T., Ohashi Y., Sugano N. (1994). Productions of interferon-gamma and nitrite are induced in mouse splenic cells by a heteroglycan-protein fraction from culture-medium of *lentinus edodes* mycelia. Immunopharmacology.

[4] Jiao J.L., Zhang M.S., Fang Y.K., Ning A.H., Zhong M.T., Huang M. (2018). Anti-tumor effect of LP1 from lentinus edodes C91-3 in mice bearing hepatic carcinoma H22 cells. Chin. J. Microecol..

[5] Barać M.B., Stanojević S.P., Jovanović S.T., Pešić M.B. (2004). Soy protein modification: A review. Atca Period. Technol..

[6] Heck T., Faccio G., Richter M., Thöny-Meyer L. (2013). Enzyme-catalyzed protein crosslinking. Appl. Microbiol. Biotechnol..

[7] Li J.X., Feng Y., Cheng Q.Y., Liu J.Y., Yun S.J., Cheng Y.F., Cheng F.E., Cao J.L., Feng C.P. (2023). Investigation of consequences of high-voltage pulsed electric field and TGase cross-linking on the physicochemical and rheological properties of *Pleurotus eryngii* protein. Foods.

[8] Xu Y.J., Zhao X., Bian G.L., Yang L., Han M.Y., Xu X.L., Zhou G.H. (2018). Structural and solubility properties of pale, soft and exudative (PSE)-like chicken breast myofibrillar protein: Effect of glycosylation. LWT.

[9] Lin G., Yuan D., Wang Q., Li W., Cai J., Li S., Lamikanra O., Qin X. (2018). Maillard-reaction-functionalized egg ovalbumin stabilizes oil nanoemulsions. J. Agric. Food Chem..

[10] Wang Q., Ismail B. (2012). Effect of Maillard-induced glycosylation on the nutritional quality, solubility, thermal stability and molecular configuration of whey protein. Int. Dairy J..

[11] Mengíbar M., Miralles B., Heras Á. (2017). Use of soluble chitosans in Maillard reaction products with β-lactoglobulin. Emulsifying and antioxidant properties. LWT.

[12] Lin J., Guo X., Ai C., Zhang T., Yu S. (2020). Genipin crosslinked sugar beet pectin-whey protein isolate/bovine serum albumin conjugates with enhanced emulsifying properties. Food Hydrocoll..

[13] Macierzanka A., Bordron F., Rigby N.M. (2011). Transglutaminase cross-linking kinetics of sodium caseinate is changed after emulsification. Food Hydrocoll..

[14] Hrynets Y., Ndagijimana M., Betti M. (2014). Transglutaminase-catalyzed glycosylation of natural actomyosin (NAM) using glucosamine as amine donor: Functionality and gel microstructure. Food Hydrocoll..

[15] Gottardi D., Hong P.K., Ndagijimana M., Betti M. (2014). Conjugation of gluten hydrolysates with glucosamine at mild temperatures enhances antioxidant and antimicrobial properties. LWT.

[16] Yang M., Shi Y., Liang Q. (2016). Effect of microbial transglutaminase crosslinking on the functional properties of yak caseins: A comparison with cow caseins. Dairy Sci. Technol..

[17] Abdelrahee A.N., Hashim A.S., Ahmed M.E.S. (2010). Changes in functional properties by transglutaminase cross linking as a function of pH of legumes protein isolate. Innov. Rom. Food Biotechnol..

[18] Black C., Clar C., Henderson R., MacEachern C., McNamee P., Quayyum Z., Thomas S. (2009). The clinical effectiveness of glucosamine and chondroitin supplements in slowing or arresting progression of osteoarthritis of the knee: A systematic review and economic evaluation. Health Technol. Asses..

[19] Chung Y.C., Tsai C.F., Li C.F. (2006). Preparation and characterization of water-soluble chitosan produced by Maillard reaction. Fish. Sci..

[20] Fan H.B., Zou Y., Huang S.Y., Liu Y.L., Zheng Q.W., Guo L.Q., Lin J.F. (2021). Study on the physicochemical and emulsifying property of proteins extracted from *Pleurotus tuoliensis*. LWT.

[21] Chelh I., Gatellier P., Santé-Lhoutellier V. (2006). A simplified procedure for myofibril hydrophobicity determination. Meat Sci..

[22] Qian J.Y., Ma L.J., Wang L.J., Jiang W. (2016). Effect of pulsed electric field on structural properties of protein in solid state. LWT.

[23] Song X.Z., Zhou C.J., Fu F., Chen Z.L., Wu Q.L. (2013). Effect of high-pressure homogenization on particle size and film properties of soy protein isolate. Ind. Crop. Prod..

[24] Miao F., Xin H.Z. (2017). Modified properties of a glycated and cross-linked soy protein isolate by transglutaminase and an oligochitosan of 5 kDa. J. Sci. Food Agric..

[25] Simonian M.H., Smith J.A. (2006). Spectrophotometric and colorimetric determination of protein concentration. Curr. Protoc. Mol. Biol..

[26] Amza T., Balla A., Tounkara F., Man L., Zhou H.M. (2013). Effect of hydrolysis time on nutritional, functional and antioxidant properties of protein hydrolysates prepared from gingerbread plum (Neocarya macrophylla) seeds. Int. Food Res. J..

[27] Mao X., Hua Y. (2012). Composition structure and functional properties of protein concentrates and isolates produced from walnut (Juglans regia L.). Int. J. Mol. Sci..

[28] Wen X.Y., Jin F., Joe M. (2018). Regenstein and Fengjun Wang, Transglutaminase induced gels using bitter apricot kernel protein: Chemical, textural and release properties. Food Biosci..

[29] Chen L., Ullah N., Li C.Y., Hackman R.M., Li Z.X., Xu X.L., Zhou G.H., Feng X.C. (2017). Incorporated glucosamine adversely affects the emulsifying properties of whey protein isolate polymerized by transglutaminase. J. Dairy Sci..

[30] van Teeffelen A.M., Broersen K., de Jongh H.H. (2005). Glucosylation of blactoglobulin lowers the heat capacity change of unfolding; a unique way to affect protein thermodynamics. Protein Sci..

[31] Mu L.X., Zhao H.F., Zhao M.M., Cui C., Liu L.Y. (2011). Physicochemical properties of soy protein isolates-acacia gum conjugates. Czech J. Food Sci..

[32] Abhishek S. (2008). Fluorescence study of the curcumin-casein micelle complexation and its application as a drug nanocarrier to cancer cells. Macromolecules.

[33] Yu J., Wang G., Wang X., Xu Y., Chen S., Wang X., Jiang L. (2018). Improving the freeze-thaw stability of soy protein emulsions via combing limited hydrolysis and Maillard-induced glycation. LWT.

[34] Spotti M.J., Martinez M.J., Pilosof A.M.R., Candioti M., Rubiolo A.C., Carrara C.R. (2014). Rheological properties of whey protein and dextran conjugates at different reaction times. Food Hydrocoll..

[35] Song C.L., Zhao X.H. (2014). Structure and property modification of an oligochitosan-glycosylated and crosslinked soybean protein generated by microbial transglutaminase. Food Chem..

[36] Liu G., Tu Z., Wang H., Zhang L., Huang T., Ma D. (2017). Monitoring of the functional properties and unfolding change of ovalbumin after DHPM treatment by HDX and FTICR MS: Functionality and unfolding of oval after DHPM by HDX and FTICR MS. Food Chem..

[37] Kong J., Yu S. (2007). Fourier transform infrared spectroscopic analysis of protein secondary structures. Acta Biochim. Biophys. Sin..

[38] Kudre T.G., Benjakul S., Kishimura H. (2013). Comparative study on chemical compositions and properties of protein isolates from mung bean black, bean and bambara groundnut. J. Sci. Food Agric..

[39] Mu L., Zhao M., Yang B., Zhao H., Cui C., Zhao Q. (2010). Effect of ultrasonic treatment on the graft reaction between soy protein isolate and gum acacia and on the physicochemical properties of conjugates. J. Agric. Food Chem..

[40] Zhang W., Leong S., Zhao F., Zhao F., Yang T., Liu S. (2018). Viscozyme L pretreatment on palm kernels improved the aroma of palm kernel oil after kernel roasting. Food Res. Int..

[41] Pinto M.S., Léonil J., Henry G., Cauty C., Carvalho A.F., Bouhallab S. (2014). Heating and glycation of β-lactoglobulin and β-casein: Aggregation and in vitro digestion. Food Res. Int..

[42] Ma Q.P., Wang H., Tu Z.C., Wen P.W., Hu Y.M. (2021). Effects of ultrasound-assisted glycation on the allergenicity of β-lactoglobulin during digestion. Food Mach..

[43] Djoullah A., Krechiche G., Husson F., Saurel R. (2016). Size measuring techniques as tool to monitor pea proteins intramolecular crosslinking by transglutaminase treatment. Food Chem..

[44] Zou P.R., Hu F., Ni Z.J., Zhang F., Thakur K., Zhang J.G., Wei Z.J. (2022). Effects of phosphorylation pretreatment and subsequent transglutaminase cross-linking on physicochemical, structural, and gel properties of wheat gluten. Food Chem..

[45] Hu X., Hu W.X., Lu H.Y., Liu S., Rao S.Q., Yang Z.Q., Jiao X.A. (2023). Glycosylated cross-linked ovalbumin by transglutaminase in the presence of oligochitosan: Effect of enzyme action time and enhanced functional properties. Food Hydrocoll..

[46] Zhang F., Cai X., Ding L., Wang S. (2021). Effect of pH, ionic strength, chitosan deacetylation on the stability and rheological properties of O/W emulsions formulated with chitosan/casein complexes. Food Hydrocoll..

[47] Bönisch M.P., Huss M., Weitl K., Kulozik U. (2007). Transglutaminase cross-linking of milk proteins and impact on yoghurt gel properties. Int. Dairy J..

[48] Boostani S., Aminlari M., Moosavi-Nasab M., Niakosari M., Mesbahi G. (2017). Fabrication and characterisation of soy protein isolate-grafted dextran biopolymer: A novel ingredient in spray-dried soy beverage formulation. Int. J. Biol. Macromol..

[49] Ter H.R., Schols H.A., Gruppen H. (2011). Effect of saccharide structure and size on the degree of substitution and product dispersity of α-lactalbumin glycated via the Maillard reaction. J. Agric. Food Chem..

[50] Hosseinzadeh H. (2010). Ceric-initiated free radical graft copolymerization of acrylonitrile onto kappa carrageenan. J. Appl. Polym Sci..

[51] Jiang S.J., Zhao X.H. (2011). Transglutaminase-induced cross-linking and glucosamine conjugation of casein and some functional properties of the modified product. Int. Dairy J..

[52] Resendiz-Vazquez J.A., Ulloa J.A., Urias-Silvas J.E., Bautista-Rosales P.U., Ramirez-Ramirez J.C., Rosas-Ulloa P., Gonzalez-Torres L. (2017). Effect of high-intensity ultrasound on the technofunctional properties and structure of jackfruit (Artocarpus heterophyllus) seed protein isolate. Ultrason. Sonochem..

[53] Li B., Bao Z., Xu W., Chi Y. (2015). Influence of glycation extent on the physicochemical and gelling properties of soybean β-conglycinin. Eur. Food Res. Technol..

[54] Tang C.H., Sun X., Foegeding E.A. (2011). Modulation of physicochemical and conformational properties of kidney bean vicilin (phaseolin) by glycation with glucose: Implications for structure-function relationships of legume vicilins. J. Agric. Food Chem..

[55] Wang Z., Han F., Sui X., Qi B., Yang Y., Zhang H., Wang R., Li Y., Jiang L. (2016). Effect of ultrasound treatment on the wet heating Maillard reaction between mung bean (Vigna radiate L.) protein isolates and glucose and on structural and physico-chemical properties of conjugates. J. Sci. Food Agric..

[56] Kihlberg J., Elofsson M. (1997). Solid-phase synthesis of glycopeptides: Immunological studies with T cell stimulating glycopeptides. Curr. Med. Chem..

[57] Liu G., Zhong Q. (2012). Glycation of whey protein to provide steric hindrance against thermal aggregation. J. Agric. Food Chem..

[58] Song C., Sun X., Yang J., Ren J., Vardhanabhuti B., Liu X., Fu Y. (2021). TGase-induced glycosylated soy protein products with limited enzymatic hydrolysis showed enhanced foaming property. Eur. Food Res. Technol..

[59] Song Y., Babiker E.E., Usui M., Saito A., Kato A. (2002). Emulsifying properties and bactericidal action of chitosan–lysozyme conjugates. Food Res. Int..

[60] Anuradha S.N., Prakash V. (2009). Altering functional attributes of proteins through cross linking by transglutaminase–a case study with whey and seed proteins. Food Res. Int..

[61] Zhu C.Y., Wang X.P., Zhao X.H. (2015). Property modification of caseinate responsible to transglutaminase-induced glycosylation and crosslinking in the presence of a degraded chitosan. Food Sci. Biotechnol..

[62] Gordon M.H. (1993). World oilseeds: Chemistry, technology, and utilization. Food Chem..

[63] Matemu A.O., Kayahara H., Murasawa H., Nakamura S. (2009). Importance of size and charge of carbohydrate chains in the preparation of functional glycoproteins with excellent emulsifying properties from tofu whey. Food Chem..

[64] Ren J., Song C., Wang P., Li S., Zheng X.Q. (2015). Modification of structural and functional properties of sunflower 11S globulin hydrolysates. Czech J. Food Sci..

[65] Lv L., Chi Y., Chen C., Xu W. (2015). Structural and functional properties of ovalbumin glycated by dry-heating in the presence of maltodextrin. Int. J. Food Prop..

